# Concurrent hippocampal induction of MHC II pathway components and glial activation with advanced aging is not correlated with cognitive impairment

**DOI:** 10.1186/1742-2094-8-138

**Published:** 2011-10-11

**Authors:** Heather D VanGuilder, Georgina V Bixler, Robert M Brucklacher, Julie A Farley, Han Yan, Junie P Warrington, William E Sonntag, Willard M Freeman

**Affiliations:** 1 Department of Pharmacology, Pennsylvania State University College of Medicine, 500 University Drive, Hershey, Pennsylvania, 17057, USA; 2 Donald W. Reynolds Department of Geriatric Medicine, Reynolds Oklahoma Center on Aging, University of Oklahoma Health Sciences Center, 975 NE 10th Street, BRC-1303, Oklahoma City, Oklahoma, 73104, USA

**Keywords:** hippocampus, cognitive decline, para-inflammation, neuroinflammation, aging, Morris water maze

## Abstract

**Background:**

Age-related cognitive dysfunction, including impairment of hippocampus-dependent spatial learning and memory, affects approximately half of the aged population. Induction of a variety of neuroinflammatory measures has been reported with brain aging but the relationship between neuroinflammation and cognitive decline with non-neurodegenerative, normative aging remains largely unexplored. This study sought to comprehensively investigate expression of the MHC II immune response pathway and glial activation in the hippocampus in the context of both aging and age-related cognitive decline.

**Methods:**

Three independent cohorts of adult (12-13 months) and aged (26-28 months) F344xBN rats were behaviorally characterized by Morris water maze testing. Expression of MHC II pathway-associated genes identified by transcriptomic analysis as upregulated with advanced aging was quantified by qPCR in synaptosomal fractions derived from whole hippocampus and in hippocampal subregion dissections (CA1, CA3, and DG). Activation of astrocytes and microglia was assessed by GFAP and Iba1 protein expression, and by immunohistochemical visualization of GFAP and both CD74 (Ox6) and Iba1.

**Results:**

We report a marked age-related induction of neuroinflammatory signaling transcripts (*i.e*., MHC II components, toll-like receptors, complement, and downstream signaling factors) throughout the hippocampus in all aged rats regardless of cognitive status. Astrocyte and microglial activation was evident in CA1, CA3 and DG of intact and impaired aged rat groups, in the absence of differences in total numbers of GFAP^+ ^astrocytes or Iba1^+ ^microglia. Both mild and moderate microglial activation was significantly increased in all three hippocampal subregions in aged cognitively intact and cognitively impaired rats compared to adults. Neither induction of MHCII pathway gene expression nor glial activation correlated to cognitive performance.

**Conclusions:**

These data demonstrate a novel, coordinated age-related induction of the MHC II immune response pathway and glial activation in the hippocampus, indicating an allostatic shift toward a para-inflammatory phenotype with advancing age. Our findings demonstrate that age-related induction of these aspects of hippocampal neuroinflammation, while a potential contributing factor, is not sufficient by itself to elicit impairment of spatial learning and memory in models of normative aging. Future efforts are needed to understand how neuroinflammation may act synergistically with cognitive-decline specific alterations to cause cognitive impairment.

## Background

Cognitive aging, characterized by a decline in a range of cognitive functions central to independence and quality of life, affects more than half of the population over 60 years of age [1]. Spatial learning and memory is one of the domains of cognitive function most frequently and severely impacted with aging [2]. Spatial cognitive function is mediated, to a large extent, by the hippocampus, which undergoes numerous molecular and physiological changes with aging. These alterations include vascular rarefaction, decreased trophic support, decreased glucose utilization and bioenergetic metabolism, and impaired protein synthesis and quality control (reviewed in [3]). Additionally, with advancing age, hippocampal volume decreases and neurotransmission and synaptic integrity decline, all in the absence of gross neuronal loss or overt neuropathology [4-9]. The molecular and cellular basis of these changes may include misfolded proteins and protein aggregates [10], synaptic pruning [11], decreased synaptic protein expression [12], and increased oxidative stress [8], which together suggest that the neural microenvironment becomes dysregulated in the aged hippocampus. This dysregulation may indicate a declining ability of glial cells to perform their roles in debris clearance, nutritional support, and even neurotransmission, which are vital for maintenance of hippocampal function and hippocampus-dependent spatial learning and memory [13-16].

The glial shift toward an activated phenotype with normal aging likely reflects increased inflammatory signaling, which has been implicated in damage- and disease-related cognitive impairment as discussed below. Pathological gliosis and inflammation are associated with severe cognitive dysfunction in neurodegenerative/advanced disease states (*e.g.*, Alzheimer's disease, vascular dementia), traumatic brain injury, chronic stress and direct inflammatory stimulation (*e.g.*, lipopolysaccharide injection, transgenic manipulation) [17-24]. Deficits of hippocampus-dependent cognitive function with healthy aging are less severe and more heterogeneous, affecting a subset of the aging population while others retain normal cognitive capabilities. Rodent models of normative human aging reflect this behavioral heterogeneity, which enables segregation of aged animals into cognitively intact and cognitively impaired groups and assessment of both age-related and cognitive impairment-specific phenomena [25-27]. Glial activation and induction of inflammatory response factors are recognized components of normal brain aging, but characterization of hippocampal cellular and molecular mediators of immune/inflammatory signaling in cognitively stratified subjects remains incomplete. Studies of severe neurodegenerative conditions characterized by significant neuronal loss suggest that neuroinflammation is a causative factor in cognitive impairment [28,29]. The relationship between neuroinflammatory signaling and non-neurodegenerative age-related cognitive decline, however, is not understood, and is likely less straightforward than that observed with neurodegenerative conditions.

Here, we demonstrate the age-related induction of 21 inflammation-response genes including MHC II antigen processing components, antigen-recognizing receptor pathways, immune cell activating factors, and downstream inflammatory signaling molecules in whole-hippocampus synaptosomal fractions and in discrete hippocampal subregions (CA1, CA3, DG) derived from independent cohorts of rats behaviorally assessed for hippocampus-dependent learning and memory by Morris water maze testing. MHC II signaling has a pivotal role in immune responses and inflammation, responding to both exogenous (*e.g.*, bacterial) and endogenous (*e.g*., protein aggregates, necrotic cell debris) antigenic proteins. These antigenic proteins bind to molecules including toll-like receptors (*Tlr2*, *Tlr4*, *Tlr7*) and complement components (*C1s*, *C3*, *C4a*, *Serping1 *[*C1inh*]), which recognize potentially threatening peptide sequences classified as PAMPs (pathogen-associated molecular patterns) and DAMPs (danger-associated molecular patterns). Recognition of these sequences stimulates immune cell-activating factors (*Erbb3*, *Ccr5*, *Fcgr2a*, *Fcgr2b*) and internalization of the protein, processing, and subsequent presentation by MHC II (for review see [30]and [31]). The MHC II complex consists of alpha and beta chains (*Hla-Dra *and *Hla-Drb*), which heterodimerize to form an antigen binding pocket. This pocket is typically blocked by a cathepsin (*Ctse*)-cleaved peptide product of *Cd74*, called CLIP (*i.e*., the MHC II invariant chain), which prevents spontaneous binding of self-derived peptides. An MHC II cofactor consisting of its own alpha and beta chain subunits (*Hla-Dm*) facilitates removal of CLIP and loading of the antigenic peptide prior to MHC II trafficking to the membrane for presentation to immune-response cells.

In the central nervous system, microglia constitute the primary line of immunity and defense, and as such, are the primary mediators of MHC II antigen processing and presentation, whereas MHC II is typically expressed only at nominal levels in astrocytes *in vivo *[32,33]. Toll-like receptors and complement components, as well as downstream inflammatory signaling factors (*Hsbp1, Lgals3, Cp, Icam1, S100a6*), on the other hand, are more broadly expressed by astrocytes, microglia and neurons In the CNS, both microglia and astrocytes play regulatory and supportive roles in neuronal function by metabolizing glutamate, providing nutritional support, and removing potentially toxic cell debris [15,34,35] In the aged hippocampus, cellular debris detected by activated microglia and astrocytes may include degenerating synaptic terminals [5,11,36], myelin fragments [37], and misfolded proteins [10].

The goal of this study was to examine MHC II pathway gene expression and glial activation measures in adult and aged, cognitively stratified animals to determine the relationship of these neuroinflammatory measures to cognitive decline. Despite the widely accepted concept of increased glial activation and MHC II induction with aging, quantitative assessments of hippocampal microglial activation (percentage of total microglial activated) and comprehensive assessment of MHC II gene expression have not been performed in behaviorally characterized adult and aged animals. Additionally, the distribution these neuroinflammatory measures across hippocampal subregions has not been examined. This work demonstrates for the first time that induction of the MHC II antigen processing and presentation pathway with aging occurs concomitantly with glial activation, and involves upregulation of complement and toll-like receptors as well as downstream inflammation response factors. Our findings also indicate that induction of hippocampal neuroinflammation, while a potential contributing factor to cognitive decline, does not in itself manifest in age-related impairment of spatial learning and memory.

## Methods

### Animals

Male Fischer 344 × Brown Norway (F1) hybrid rats (see Table 1 for cohort information) were purchased from the Harlan Industries (Indianapolis, IN) National Institute on Aging colony as previously described [27] and quarantined/acclimatized for two weeks upon arrival. Rats were singly housed in laminar flow cages with free access to food (Purina Mills, Richmond, IN) and water and maintained on a 12-hour light/dark cycle with constant temperature and humidity in the OUHSC specific pathogen-free Barrier Facility. One week after completion of behavioral testing, rats were sacrificed by decapitation without anesthesia, and the hippocampi rapidly dissected for synaptosome preparation (set 1) or hippocampal subregion dissection (set 2). Alternatively, rats were perfusion-fixed, and their brains extracted for immunohistochemistry (set 3). At sacrifice, animals were examined for exclusionary criteria (e.g., kidney disease, cardiac hypertrophy, peripheral tumors, pituitary tumors, cortical atrophy). The OUHSC animal facilities are fully accredited by the Association for Assessment and Accreditation of Laboratory Animal Care, and all animal procedures were approved by the Institutional Animal Care and Use Committee in compliance with the Public Health Service Policy on Humane Care and Use of Laboratory Animals and the National Research Council's Guide for the Care and Use of Laboratory Animals. The three independent animal cohorts used in this study are summarized in Table 1.

**Table 1:** Animal cohort information

Cohort	Age (months)	Group	n	Sample Type	Analyses Performed
1*	12	Adult	5		
			
	28	Aged Intact	8	synaptosomes (whole hippocampus)	Morris water maze, transcriptomic analysis, qPCR
			
	28	Aged Impaired	7		

2	12	Adult	7		
			
	26	Aged Intact	7	Hippocampal subregions (CA1, CA3, DG)	Morris water maze, qPCR, immunoblotting
			
	26	Aged Impaired	10		

3*	13	Adult	3		
			
	26	Aged Intact	3	Perfusion-fixed sagittal brain sections	Morris water maze, immunohistochemistry
			
	26	Aged Impaired	3		

* Described in reference 27

### Morris water maze testing

Rats were acclimatized to the OUHSC Barrier Facility for two weeks prior to hippocampus-dependent spatial learning and memory assessment conducted as previously described [27,38]. Briefly, a water maze (1.7 m × 0.6 m) was filled to a depth of 25 cm with water made opaque with non-toxic, water-based white food coloring, and a retractable 12 cm^2 ^escape platform was fixed 2 cm beneath the water's surface. A curtain with fixed-position visual cues, serving as reference cues for the location of the escape platform, surrounded the maze pool. A center-mounted camera provided visual input to an automated tracking system (Noldus Ethovision XT, Wageningen, Netherlands) for evaluation of maze performance. Task acquisition was conducted over eight days, in two-day blocks consisting of five 60 s trials each. The submerged escape platform position was fixed throughout acquisition. Path length to find the platform was the dependent measure, with shorter path lengths indicating better performance. After completion of each acquisition block (i.e., on days 2, 4, 6 and 8), a 30 s probe trial was performed with the escape platform removed. Rats were placed into the maze and the mean proximity to the platform location, duration in the annulus-40 (the area 40 cm around the platform location), cumulative distance, and mean swim velocity were recorded. To avoid extinguishing memory of the platform location, the platform was then replaced and rats were given an additional 60 s to locate it using the surrounding cues. Two days following conclusion of water maze testing, visual performance was assessed over four consecutive swim trials with the escape platform visible to ensure that maze performance was not affected by visual deficits.

Probe trial data were used to segregate aged animals into cognitively intact and impaired groups relative to the performance of adult rats, allowing retrospective analysis of acquisition phase data by group. As previously described [27], mean proximity to the escape platform location was used as the primary measure of cognitive performance on probe trials based on demonstration of its superior sensitivity compared to alternative measures [39]. The number of cumulative platform location crossings was used as a secondary measure of cognitive performance [40]. For descriptive purposes mean probe trial proximity-to-platform values of retrospectively stratified groups were assessed by one-way ANOVA with Student Newman Keuls (SNK) post hoc testing to confirm that the performance of the aged, cognitively impaired group was indeed inferior to the adult and aged, cognitively intact groups. To ascertain successful learning of the task by probe performance-stratified groups, acquisition data were statistically analyzed across blocks by one-way repeated measures ANOVA with Holm-Sidak post hoc testing. Significance of group differences for individual acquisition blocks and probe trials was assessed by one-way ANOVA with Student Newman Keuls post hoc testing.

### Synaptosome isolation

Hippocampal synaptosomes were prepared as previously described [12,27]. Briefly, hippocampi were rapidly dissected into ice-cold HEPES-buffered sucrose (320 mM sucrose, 4 mM HEPES, 1 mM, Na_3_VO_4_, pH 7.4) and incubated on ice for 30 min with buffer replaced twice at 10 minute intervals. Hippocampi were homogenized in 8 mL buffered sucrose with a mechanically-driven dounce and nuclear, cytoskeletal, and synaptosomal fractions were separated by differential centrifugation. Synaptosome samples were then resuspended in Tri-Reagent for subsequent RNA extraction.

### Hippocampal subregion dissection

For dissection of hippocampal subregions of interest (CA1, CA3, DG), left and right hippocampi were individually hemisected and the dorsomedial portion was further dissected into four blocks perpendicular to the longitudinal axis. From these blocks, the CA3 was dissected by cutting along the edge of the DG and the CA1 and DG were dissected by cutting along the hippocampal fissure as described previously [41].

### Perfusion fixation and embedding

Rats used for immunohistochemical assessment were anesthetized with ketamine/xylazine and transcardially perfused with 6U/mL heparin (sodium salt) in PBS followed by phosphate-buffered 4% paraformaldehyde (pH 7.4). Brains were extracted and hemisected sagittally, immersion-fixed in 4% paraformaldehyde (pH 7.4) overnight at 4°C, rinsed twice in PBS, and impregnated with 30% sucrose as previously described [27]. Tissue samples were embedded in Tissue-Tek optimal cutting temperature compound (Sakura Finetek, Torrance, CA, USA), frozen in isopentane on dry ice, and stored at -80°C.

### RNA isolation

Hippocampal synaptosome and dissected subregion samples were homogenized in 300 μL TriReagent (Molecular Research Center, Cincinnati, OH) by bead mill (Retsch TissueLyzer II, Qiagen, Valencia CA, USA) as previously described [27]. RNA was isolated from synaptosomal homogenates by addition of 10% BCP and standard phase separation, followed by overnight isopropanol precipitation at -20°C. RNA was purified using the Qiagen RNeasy Mini kit (Qiagen), and quality and quantity were assessed by microfluidics chip (Agilent 2100 Expert Bioanalyzer Nano Chip, Agilent, Palo Alto, CA) and spectrometry (NanoDrop ND1000; Thermo Scientific, Wilmington, DE), respectively, with RNA integrity numbers > 8 used as exclusion criteria.

### Microarray analysis

Transcriptomic analysis of hippocampal synaptosomes derived from adult, aged intact, and aged impaired rats (n = 5-7/group) was performed using Illumina RatRef-12 microarrays (Illumina, San Diego, CA) according to standard methods and as previously described [42,43]. Briefly, first-strand cDNA was synthesized from 500 ng input RNA by two-hour incubation at 42°C with T7 Oligo(dT) primer, 10 × First Strand buffer, dNTPs, RNase inhibitor, and ArrayScript. Second-strand cDNA was synthesized from first-strand cDNA by two hour incubation at 16°C with 10 × Second Strand buffer, dNTPs, DNA polymerase, and RNase H, purified using the Illumina TotalPrep kit (Ambion, Foster City, CA) according to the manufacturer's protocols and eluted in 19 μL 55°C nuclease-free water. cRNA was synthesized from second-strand cDNA using the MEGAscript kit (Ambion), and labelled by incubation for 14 hours at 37°C with T7 10× Reaction Buffer, T7 Enzyme mix, and Biotin-NTP mix. Following purification with the Illumina TotalPrep RNA Amplification kit (Ambion) according to manufacturer's instructions, cRNA yields were quantitated using a NanoDrop ND1000 spectrometer. Biotinylated cRNA (750 ng) was hybridized to RatRef-12 BeadChips by incubating for 20 hours at 58°C at a rocker speed of 5. After incubation, BeadChips were washed and streptavidin-Cy3 stained, dried by centrifugation at 275 × g for 4 min and scanned and digitized using a Bead Station Bead Array Reader.

Arrays were quality control checked, and initial data analysis using average normalization with background subtraction was performed in GenomeStudio software (Illumina). The full microarray dataset has been deposited in the Gene Expression Omnibus, accession# (GSE29511). Using detection p-values generated by GenomeStudio, probes were filtered for only those with present or marginal calls in 100% of the samples in at least one of the three experimental groups. This ensured that transcripts not reliably detected in the experiment were excluded from statistical analysis, and that genes potentially expressed in only one experimental animal group (i.e., in adult, aged intact or aged impaired rats only) were retained. Statistically significant differential gene expression was determined using a combination of pair-wise absolute value fold-change cutoff of 1.2 and one-way ANOVA with Student Newman Keuls post hoc testing p < 0.05 [43].

### Bioinformatic analysis and visualization

Array data were imported into Ingenuity Pathway Analysis software (Ingenuity Systems, Redwood City, CA) for determination of significantly regulated pathways/networks (Fisher's Exact Test, p < 0.001) represented by genes differentially expressed between groups. The distribution of gene expression for the regulated pathway of interest was visualized by a heatmap generated in GeneSpring GX11 (Agilent) with hierarchical clustering by individual samples and genes using average distance and complete linkage. Gene expression levels represented on the heatmap were log-scaled to the adult mean expression per gene, with green < 1.0, black = 1.0, and red > 1.0, with color hue indicative of degree of down- or up-regulation.

### RT-qPCR

Confirmation of gene expression levels was performed as previously described using whole-hippocampus synaptosomes (n = 5-8/group) with assessments expanded to individual hippocampal subregions (n = 7-10/group). cDNA was synthesized from purified RNA with the ABI High-capacity cDNA Reverse Transcription kit (Applied Biosystems, Foster City, CA). For each sample, 1 μg RNA was reacted with random primers, dNTPs, and MultiScribe Reverse Transcriptase enzyme using a GeneAmp PCR 9700 System (Applied Biosystems), as previously described [27,43,44]. qPCR analysis of targets of interest was performed using standard laboratory methods and TaqMan Assay-On-Demand (Applied Biosystems, Foster City, CA, USA) gene-specific primers/probe assays (Table 2) and a 7900HT Sequence Detection System (Applied Biosystems) [27,43,44]. Relative gene expression was calculated with SDS 2.2.2 software using the 2^-ΔΔCt ^analysis method with β-actin as an endogenous control. Statistical analysis of age-related (*i.e.*, adult vs. aged) gene expression changes in microarray-confirmation qPCR experiments was performed by two-tailed t-testing with Benjamini-Hochberg multiple testing correction (BHMTC). To assess the potential regulation of mRNA expression with cognitive status (adult, aged cognitively intact, and aged cognitively impaired), qPCR data were analyzed by one-way ANOVA with BHMTC, followed by SNK post hoc testing of pair-wise comparisons. Potential relationships between gene expression and behavioral performance (mean proximity to platform) were assessed by Pearson product moment correlation analyses with BHMTC.

**Table 2:** Primer/probe information

Gene Symbol	Gene ID	Gene Name	TaqMan AOD #
C1s	192262	complement component 1, subcomponent s	Rn00594278_m1

C3	24232	complement component 3	Rn00566466_m1

C4a	24233	complement component 4a	Rn00709527_m1

Ccr5	117029	chemokine (C-C motif) receptor 5	Rn00588629_m1

Cd74	25599	CD74 molecule, major histocompatibility complex, class II invariant chain	Rn00565062_m1

Cp	24268	ceruloplasmin	Rn00561049_m1

Ctse	25424	cathepsin E	Rn00564036_m1

Erbb3	29496	v-erb-b2 erythroblastic leukemia viral oncogene homolog 3	Rn00568107_m1

Fcgr2a	116591	Fc fragment of IgG, low affinity IIa, receptor (CD32)	Rn00821543_g1

Fcgr2b	289211	Fc fragment of IgG, low affinity IIb, receptor (CD32)	Rn00598391_m1

Hla-dmb	3109	major histocompatibility complex, class II, DM beta	Rn01429041_m1

Hla-dra	294269	major histocompatibility complex, class II, DR alpha	Rn01427980_m1

Hla-drb1	294270	major histocompatibility complex, class II, DR beta 1	Rn01429350_m1

Hsbp1	286899	heat shock factor binding protein 1	Rn00583001_g1

Icam1	25464	intercellular adhesion molecule 1	Rn00564227_m1

Lgals3	83781	lectin, galactoside-binding, soluble, 3	Rn00582910_m1

S100a6	85247	S100 calcium binding protein A6	Rn00821474_g1

Serping1	295703	serpin peptidase inhibitor, clade G (C1 inhibitor), member 1	Rn01485600_m1

Tlr2	310553	toll-like receptor 2	Rn02133647_s1

Tlr4	29260	toll-like receptor 4	Rn00569848_m1

Tlr7	317468	toll-like receptor 7	Rn01771083_s1

### Protein Extraction

Soluble protein was isolated by homogenizing samples in a detergent-based protein lysis buffer containing protease and phosphatase inhibitors (100 mM NaCl, 20 mM HEPES, 1 mM EDTA, 1 mM dithiothreitol, 1.0% Tween20, 1 mM Na_3_VO_4_, 1 Complete Mini EDTA-Free Protease Inhibitor Cocktail Tablet (Roche Applied Science, Indianapolis, IN) for every 10 mL lysis buffer) using a bead mill. Homogenates were incubated for 15 min at 4°C with gentle rocking, insoluble protein was pelleted by centrifugation (12 min, 10, 000 × g, 4°C), and the soluble protein-containing supernatant was collected. Protein yields were determined by bicinchoninic acid quantitation (Pierce, Rockford, IL) in technical triplicates, and samples were adjusted to a concentration of 4 μg/μL in protein lysis buffer and LDS sample buffer (Invitrogen, Carlsbad, CA).

### Immunoblotting

Immunoblot analysis of GFAP and Iba1 expression was conducted using standard laboratory methods [12,27]. 10 μg of each prepared protein sample was denatured by heating to 95°C for 5 min prior to separation by SDS-PAGE using Criterion Tris-HCl precast gels (4-20% acrylamide gradient, 1 mm thick, 26 wells; BioRad, Hercules, CA, USA). To ensure equal protein content between samples, one gel containing all study samples was fixed with 10% ethanol/1% citric acid, stained with Deep Purple total protein stain according to manufacturer's instructions (GE LifeSciences), and quantitated by whole-lane digital densitometry (ImageQuant TL, Molecular Dynamics, Synnyvale, CA) as previously described [12]. For immunoblotting, SDS-PAGE separated proteins were transferred to PVDF membranes (HyBond, GE Healthcare) and blocked with 5% nonfat milk in PBS with 1.0% Tween-20 (PBST) prior to overnight incubation with primary antibodies to GFAP (1:1000) and Iba1 (1:2000) (Table 3) in blocking solution at 4°C with gentle rocking. Membranes were washed with PBST, incubated with secondary antibody (horseradish peroxidase-conugated donkey-anti-rabbit IgG, 1:2500), and developed with enhanced chemiluminescence substrate (GE Healthcare). Immunoreactive bands were imaged on film, digitized at a resolution of 800 dpi, and quantitated using automated digital densitometry software (ImageQuant TL). Immunoblot data were normalized to corresponding whole-lane densitometric volumes of the total protein stained gel. Pairwise comparisons (i.e., adult vs. aged groups) were assessed by two-tailed t-tests.

**Table 3:** Antibody information

Target	Supplier	Catalog #	Host	Use	Method*
Glial fibrillary acidic protein (GFAP)	Abcam	7620	rabbit	primary	IB/IHC

Ionized calcium binding adaptor molecule 1 (Iba1)	Wako Pure Chemical Industries	1620001	rabbit	primary	IB

Ionized calcium binding adaptor molecule 1 (Iba1)	Wako Pure Chemical Industries	1919741	rabbit	primary	IHC

MHC2 invariant chain (CD74; Ox6)	Abcam	23990	mouse	primary	IHC

Rabbit IgG (HRP-conjugated)	GE Healthcare	NA934V	donkey	secondary	IB

Rabbit [F(ab')_2_]	Jackson ImmunoResearch	711496152	donkey	secondary	IHC

Mouse [F(ab')_2_]	Jackson ImmunoResearch	715486150	goat	secondary	IHC

* IB: immunoblotting; IHC: immunohistochemistry

### Immunohistochemistry

Three rats from each group (adult, aged intact, aged impaired) were included in this analysis. Tissues were cryosectioned (12 μm) in the sagittal plane (HN 505E, Microm International, Walldorf, Germany) at -19°C, and sections were collected on glass slides (FisherBrand SuperFrost Plus, Fisher Scientific, Pittsburg, PA). As previously described [27] sections were postfixed with 2.0% paraformaldehyde, pH 7.4, and blocked with 10% donkey serum (Jackson ImmunoResearch, WestGrove, PA) in 0.1% Triton X-100 in PBS. Sections were incubated overnight at 4°C in blocking buffer with the addition of either antibodies to Iba1 (microglia-specific marker) and CD74 (MHC II invariant chain; activation-specific microglial marker) to visualize total and activated microglia, or to GFAP to visualize astrocytes (Table 3). Negative control slides with primary antibody were included to identify potential non-specific, background immunofluorescence of tissue and secondary antibodies. Sections were washed with 0.1% Triton X-100 in PBS, incubated with affinity-purified, species-appropriate fluorescence-conjugated secondary antibodies (donkey-anti-rabbit DyLight 649, 1:200, or donkey-anti-mouse DyLight 488, 1:200; Jackson ImmunoResearch, West Grove, PA) diluted in blocking solution, and counterstained with Hoechst 33258 (5 μg/mL, Invitrogen, Carlsbad, CA). After washing, slides were coverslipped with Aqua Poly/mount (Polysciences, Warrington, PA, USA) and imaged by confocal microscopy.

Images were acquired using a confocal laser scanning microscope (Leica TCS SP2 AOBS, Exton, PA) equipped with UV-diode (Hoechst, 405 nm), argon (488 nm), and helium-neon (546 nm and 633 nm) lasers. Subregions were imaged using a 20× objective, as 8 μm series of 24 optical sections (0.3 μm step size, 1024 × 1024 pixel resolution) and presented as average projections of z-stacks. Detailed images were obtained using a 63× objective 4× digital zoom with 2 × 2 line and frame averaging. Noise reduction and background subtraction were performed using Adobe Photoshop CS4 software, with equal adjustments applied to all images of the same antibody (Adobe Systems, San Jose, CA, USA). All images were assessed to ensure that there was no signal saturation in any channel.

### Quantitation of astrocyte and microglia populations

Hippocampal subregions of interest (CA1, CA3, DG) were imaged as 8 μm stacks for quantitation in three tissue sections per animal per group (adult, aged cognitively intact, aged cognitively impaired, n = 3/group. To identify potential proliferation of astrocytes, the number of GFAP^+ ^cells per subregion of interest was quantified using ImageJ software with the cell counter plug-in (NIH, Bethesda, MD). Quantitation of total microglia (Iba1^+^, CD74^-^, red signal), mildly activated microglia (Iba1^+^, weakly CD74^+^, orange signal), and moderately activated microglia (Iba1^+^, highly CD74^+^, yellow signal) was performed using the cell counter plug-in for ImageJ. For each subregion and tissue section, the number of mildly- and moderately-activated microglia was calculated as a percentage of total microglia present in that subregion. For all quantitation experiments, the three sections per animal were treated as technical triplicates and, for each subregion, were averaged to yield either 1) the density of astrocytes per subregion (GFAP^+ ^cells per 100 μm^2^), 2) the density of microglia per subregion (Iba1^+ ^cells per 100 μm^2^), or 3) the percentage of mildly- and moderately-activated microglia. These data were analyzed for statistical significance by one-way ANOVA with SNK post hoc testing.

## Results

### Behavioral stratification of adult and aged rats by Morris water maze performance

**Figure 1:**
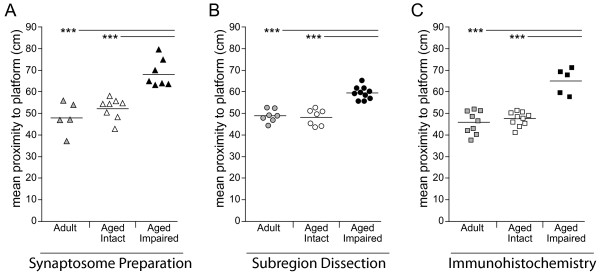
**Stratification of aged rats by cognitive performance**. Three independent cohorts of adult (12-13 months) and aged (26-28 months) male Fischer × Brown Norway hybrid (F1) rats were assessed for hippocampus-dependent spatial learning and memory using the Morris water maze. Using mean proximity to the escape platform location as the dependent variable, aged rats performing within the range of adults were classified as cognitively intact, while those with poorer performance were classified as cognitively impaired. Based on this stratification, the performance of both adult and aged intact rats was significantly superior to aged impaired rats in (A) set 1: utilized for preparation of whole-hippocampus synaptosomal fractions used in transcriptomic profiling and mRNA quantitation, (B) set 2: utilized for dissection of CA1, CA3 and DG used in mRNA and protein quantitation, and (C) set 3: perfusion-fixed for immunohistochemical assessments. Points represent individual animals and horizontal bars indicate group means; *** p < 0.001, ANOVA SNK post hoc test. See Table 1 for cohort information. Behavioral data for cohorts 1 and 3 are adapted from reference 27.

Hippocampus-dependent cognitive performance of Fischer 344 × Brown Norway hybrid (F1) male rats was assessed by Morris water maze testing, and aged animals were assigned to cognitively intact or cognitively impaired groups based on mean proximity to the escape platform during probe trials as previously described [25,27]. In all three animal cohorts used in this study, aged rats performing within the range of the adult group were classified as cognitive intact, while those with mean proximity values greater than two standard deviations above the adult group mean were classified as cognitively impaired (Figure 1). The mean probe trial performance of both adult and aged cognitively intact groups was superior to the aged impaired group, as verified by one-way ANOVA with SNK post hoc testing (p < 0.001). Retrospective analysis of acquisition phase performance demonstrated that all groups successfully learned the task, as demonstrated by decreasing path length across acquisition blocks (repeated measures ANOVA, p < 0.001). Characterization of these animals has been described, in part, elsewhere [27] and is summarized in Table 1 and Figure 1.

### Age-related upregulation of the MHC II antigen presentation pathway

**Figure 2:**
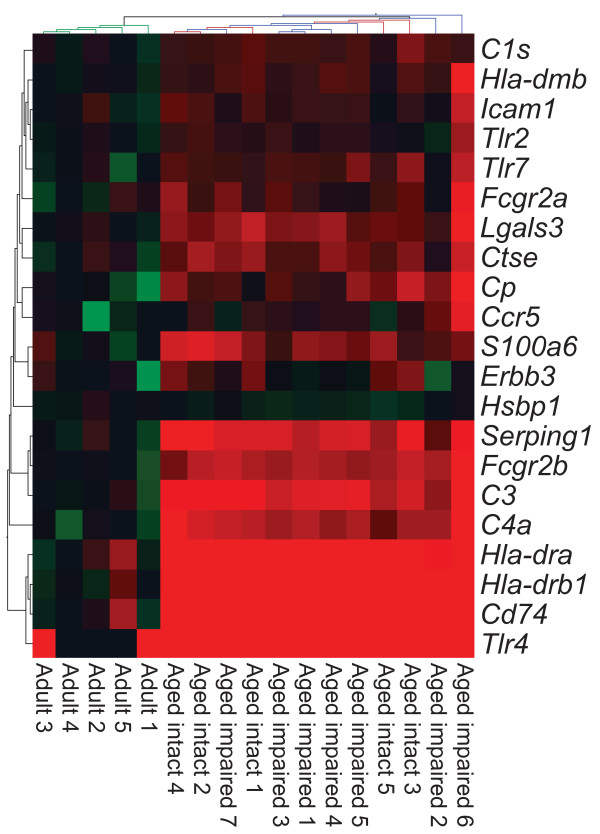
**Age-related induction of MHC II and inflammation-response transcripts**. Bioinformatic analysis of transcriptomic expression data identified significant upregulation the MHC II antigen presentation pathway and associated inflammatory signaling factors in hippocampal synaptosomes derived from both cognitively intact and cognitively impaired aged rats compared to adults (ANOVA with SNK post hoc testing, p < 0.05). Notably, no differences were observed between aged intact and aged impaired rats, which clustered separately from adults but did not cluster by cognitive status. Log-scaled gene expression is presented relative to the adult group mean per transcript (green: decreased; black: unchanged; red: increased).

Transcriptomic data obtained by microarray analysis of hippocampal synaptosomes derived from adult, aged intact, and aged impaired rats (cohort 1) were subjected to bioinformatic analysis to identify ontologies and pathways over-represented in the differentially-expressed genes. Gene Ontology (GO) analysis identified the antigen processing and presentation of exogenous antigen via MHC II (GO:019886) as over-represented among changes (Fisher's Exact Test, p < 0.002). Pathway analysis (Ingenuity) also identified the MHC II antigen presentation pathway as significantly upregulated with aging (Fisher's Exact test, p < 0.0001). Genes encoding 21 components of the MHC II machinery, antigen recognition receptors, and downstream inflammatory signaling factors (Figure 2) were significantly increased in both aged intact and aged impaired rats compared to adults. Differences in expression of these genes were not detected between aged intact and aged impaired groups. The full transcriptomic dataset is publically available [Gene Expression Omnibus, accession # (GSE29511)].

### Differential MHC II and related gene expression with aging occurs throughout the hippocampus

To confirm the transcriptomic finding that the MHC II immune pathway is upregulated with aging in hippocampal synaptosome samples and expand quantitation to individual hippocampal subregions, qPCR analysis of 21 MHC II-associated genes differentially expressed in the microarray analysis was performed. This quantitative analysis was performed using the same synaptosomal fractions assessed by microarray profiling, as well as unfractionated CA1, CA3 and DG dissections derived from an independent, behaviorally characterized animal cohort. qPCR data was first examined for age-related changes (all aged vs. adult, t-test, BHMTC) to confirm the microarray findings. qPCR data were also compared to individual animal performance data for potential significant correlations to behavioral performance (Pearson correlation, BHMTC). Additionally, a three-group comparison with the aged group split into intact and impaired groups was performed to examine potential cognition specific-effects (ANOVA, with SNK pairwise comparisons, BHMTC). Statistical results are summarized in Table 4, and group means (± S.E.M.) are presented in Table 5. All 21 genes selected from the transcriptomic data for confirmation were significantly induced with aging in the synaptosomal samples. In subregion samples the age-related induction of gene expression was most evident in CA3 and CA1, with fewer genes significantly upregulated in the DG (Table 4). MHC II component genes were the most highly induced genes, with age-related increases in expression as high as 10-fold. While direct statistical comparisons between subregions are not possible as these data were in collected independent qPCR experiments, the magnitude of changes was generally highest in synaptosomal samples, followed by CA3 and CA1. In no case did expression of these 21 age-regulated genes significantly correlate with cognitive performance. In only two instances were expression differences evident between aged impaired and aged intact groups (DG; Hla-dmb, C3) and these were of a small magnitude.

**Table 4:** qPCR confirmation of age-related induction of MHC II pathway component genes

Gene	**All Aged vs. Adult ***(ratio, p-value t-test)*				Pearson Correlation to Morris water maze				**Aged Intact vs. Adult ***(ratio, p-value SNK)*				**Aged Impaired vs. Adult***(ratio, p-value SNK)*				**Aged Impaired vs**. **Aged Intact***(ratio, p-value SNK)*			
	**Syn**	**CA1**	**CA3**	**DG**	**Syn**	**CA1**	**CA3**	**DG**	**Syn**	**CA1**	**CA3**	**DG**	**Syn**	**CA1**	**CA3**	**DG**	**Syn**	**CA1**	**CA3**	**DG**

***MHC II Components***																				

*Cd74*	6.58^###^	5.61^###^	3.61^###^	2.43^###^	-	-	-	-	6.79***	5.66***	3.88***	2.43***	6.35***	5.58***	3.40***	2.49***	-	-	-	*-*

*Ctse*	1.69^##^	1.45^##^	1.75^###^	1.48^###^	-	-	-	-	1.81*	*-*	1.73***	1.48*	*-*	*-*	1.77***	1.54**	-	-	-	*-*

*Hla-dmb*	1.64^###^	1.37^###^	1.37^###^	1.26^##^	-	-	-	-	1.57**	1.33**	1.39***	*-*	1.72***	1.40***	1.35***	1.33**	-	-	-	1.16*

*Hla-dra*	6.30^###^	4.04^###^	3.02^###^	-	-	-	-	-	6.76***	4.18***	3.25***	*-*	5.75**	3.95***	2.85***	*-*	-	-	-	*-*

*Hla-drb1*	10.50^###^	7.13^###^	4.39^###^	2.91^#^	-	-	-	-	10.70***	7.12***	4.77***	*-*	10.26***	7.13***	4.12***	3.42**	-	-	-	*-*

***Antigen Recognition***																				

*C1s*	1.84^#^	1.21^#^	1.56^###^	-	-	-	-	-	-	*-*	1.59**	-	-	*-*	1.53***	-	-	-	-	*-*

*C3*	3.47^###^	2.00^###^	1.96^###^	1.69^###^	-	-	-	-	3.40**	2.07***	2.22***	1.52***	3.55**	1.94***	1.78***	1.81***	-	-	-	1.19*

*C4a*	4.18^###^	1.77^###^	1.51^###^	-	-	-	-	-	3.83**	1.67***	1.58***	*-*	4.59**	1.85***	1.47***	*-*	-	-	-	*-*

*Serping1*	2.71^###^	2.03^###^	2.27^###^	1.54^###^	-	-	-	-	2.79***	2.18***	2.52***	1.49**	2.63***	1.92***	2.09***	1.58***	-	-	-	*-*

*Tlr2*	1.64^##^	*-*	1.54##	-	-	-	-	-	1.55*	*-*	1.66*	*-*	1.73*	*-*	1.46*	*-*	-	-	-	*-*

*Tlr4*	1.51^##^	1.35^#^	1.96^###^	-	-	-	-	-	1.41*	*-*	2.00***	*-*	1.60*	*-*	1.93***	*-*	-	-	-	*-*

*Tlr7*	1.69^##^	1.34^##^	1.73^###^	*-*	-	-	-	-	1.65*	1.34*	1.74***	*-*	1.73*	1.35*	1.72***	*-*	-	-	-	*-*

***Immune Cell Activating***																				

*Ccr5*	1.96^#^	*-*	-	*-*	-	-	-	-	-	*-*	-	*-*	*-*	*-*	*-*	*-*	-	-	-	*-*

*Erbb3*	1.28^###^	1.34^###^	1.20^#^	1.40^#^	-	-	-	-	1.26**	1.43***	-	*-*	1.30**	1.28**	*-*	1.47**	-	-	-	*-*

*Fcgr2a*	1.45^#^	1.19^#^	1.30^###^		-	-	-	-	-	*-*	1.30**	*-*	*-*	*-*	1.30**	*-*	-	-	-	*-*

*Fcgr2b*	1.96^##^	*-*	1.61^###^	*-*	-	-	-	-	2.08*	*-*	1.63***	*-*	1.82*	*-*	1.60***	*-*	-	-	-	*-*

***Inflammation Response***																				

*Cp*	1.67^#^	*-*	1.56^###^	*-*	-	-	-	-	1.92*	*-*	1.64***	*-*	*-*	*-*	1.50***	*-*	-	-	-	*-*

*Hsbp1*	2.87^###^	1.68^###^	2.09^###^	*-*	-	-	-	-	3.13**	1.65**	2.25***	*-*	2.58***	1.70**	1.97***	*-*	-	-	-	*-*

*Icam1*	1.46^#^	1.55^###^	1.43^###^	*-*	-	-	-	-	*-*	1.68***	1.64***	*-*	*-*	1.46**	1.31**	*-*	-	-	-	*-*

*Lgals3*	3.42^##^	2.64^###^	1.98^###^	1.84^##^	-	-	-	-	3.68***	2.62***	2.18***	1.63*	3.03***	2.66***	1.84***	2.00**	-	-	-	*-*

*S100a6*	1.52^#^	2.03^###^	2.21^###^	*-*	-	-	-	-	*-*	2.00***	2.48***	*-*	-	2.06***	2.02***	*-*	-	-	-	*-*

qPCR confirmation of transcriptomic findings was performed in hippocampal synaptosomes and expanded to include hippocampal subregion dissections. Age-based comparisons (aged intact and aged impaired versus adult) were statistically analyzed by two-tailed t-test with BHMTC (^#^p < 0.05; ^##^p < 0.01; ^###^p < 0.001). Three-group data (adult, aged intact, aged impaired) were statistically analyzed by one-way ANOVA with BHMTC, and pairwise comparisons were evaluated by SNK post hoc testing to identify potential regulation of gene expression with cognitive decline (*p < 0.05; **p < 0.01; ***p < 0.001). Correlation of gene expression with Morris water maze performance was assessed by Pearson product moment analysis with BHMTC, which demonstrated no significant association of MHC II pathway gene expression and cognitive impairment. Data are presented as ratios of group means. Individual group gene expression data is presented in Table 5.

**Table 5:** Gene expression levels of age and cognitive status groups, presented as group mean (% of adult mean) ± SEM

	CA1				CA3				DG				synaptosomes (whole-hippocampus)			
			***Aged***	***Aged***			***Aged***	***Aged***			***Aged***	***Aged***			***Aged***	***Aged***

*Gene*	***Adult***	***Aged***	*Intact*	*Impaired*	***Adult***	***Aged***	*Intact*	*Impaired*	***Adult***	***Aged***	*Intact*	*Impaired*	***Adult***	***Aged***	*Intact*	*Impaired*

*C1s*	**100 ± 2.8**	**121 ± 6.1**	125 ± 11.7	118 ± 6.6	**100 ± 7.8**	**156 ± 7.6**	159 ± 13.4	153 ± 9.3	**100 ± 6.1**	**122 ± 4.8**	117 ± 5.7	125 ± 6.9	**100 ± 21.7**	**184 ± 21.8**	182 ± 28.6	186 ± 35.3

*C3*	**100 ± 6.6**	**200 ± 10.9**	207 ± 16.4	194 ± 15.0	**100 ± 7.9**	**196 ± 10.8**	222 ± 17.8	178 ± 10.7	**100 ± 8.1**	**169 ± 7.2**	152 ± 10.2	181 ± 8.3	**100 ± 23.2**	**347 ± 35.7**	340 ± 27.6	355 ± 74.4

*Serping1*	**100 ± 7.4**	**203 ± 13.4**	218 ± 14.6	192 ± 20.2	**100 ± 7.3**	**227 ± 11.5**	252 ± 17.4	209 ± 13.1	**100 ± 4.8**	**154 ± 6.3**	149 ± 9.4	158 ± 8.7	**100 ± 6.9**	**271 ± 12.4**	279 ± 19.9	263 ± 15.5

*Cd74*	**100 ± 7.7**	**561 ± 33.3**	566 ± 44.5	558 ± 47.9	**100 ± 10.9**	**361 ± 20.5**	388 ± 37.6	340 ± 21.2	**100 ± 7.7**	**243 ± 17.2**	233 ± 36.7	249 ± 18.2	**100 ± 28.8**	**658 ± 48.9**	679 ± 46.7	635 ± 95.8

*C4a*	**100 ± 7.1**	**177 ± 7.0**	167 ± 11.1	185 ± 8.6	**100 ± 4.8**	**151 ± 4.6**	158 ± 6.2	147 ± 6.2	**100 ± 9.7**	**135 ± 10.3**	117 ± 15.2	147 ± 13.0	**100 ± 17.7**	**418 ± 44.6**	383 ± 31.6	459 ± 88.3

*Ccr5*	**100 ± 5.2**	**108 ± 5.1**	106 ± 5.2	110 ± 7.8	**100 ± 3.1**	**121 ± 6.3**	117 ± 7.3	125 ± 10.3	**100 ± 6.9**	**99 ± 7.0**	88 ± 7.1	106 ± 10.4	**100 ± 17.9**	**196 ± 23.0**	172 ± 18.8	220 ± 41.8

*Cp*	**100 ± 5.4**	**93 ± 8.2**	93 ± 6.9	93 ± 13.5	**100 ± 3.9**	**156 ± 6.3**	164 ± 9.2	150 ± 8.4	**100 ± 9.2**	**110 ± 8.0**	118 ± 13.7	103 ± 9.8	**100 ± 10.8**	**167 ± 17.1**	192 ± 28.6	146 ± 18.4

*Ctse*	**100 ± 8.0**	**145 ± 10.2**	141 ± 13.7	148 ± 14.4	**100 ± 9.8**	**175 ± 7.4**	173 ± 13.9	177 ± 8.7	**100 ± 6.8**	**148 ± 6.8**	140 ± 11.3	154 ± 8.4	**100 ± 21.0**	**169 ± 12.0**	181 ± 19.8	156 ± 11.4

*Erbb3*	**100 ± 2.7**	**134 ± 5.1**	143 ± 7.5	128 ± 6.5	**100 ± 8.1**	**120 ± 5.1**	128 ± 9.1	114 ± 5.6	**100 ± 7.5**	**140 ± 7.8**	129 ± 10.9	147 ± 10.3	**100 ± 5.4**	**128 ± 4.0**	126 ± 6.5	130 ± 4.6

*Fcgr2b*	**100 ± 3.2**	**108 ± 7.9**	103 ± 6.4	112 ± 13.0	**100 ± 9.0**	**161 ± 5.2**	163 ± 12.0	160 ± 3.6	**100 ± 4.2**	**124 ± 11.0**	125 ± 19.8	123 ± 13.2	**100 ± 20.8**	**196 ± 15.4**	208 ± 22.6	182 ± 21.2

*Fcgr2a*	**100 ± 4.4**	**119 ± 4.8**	116 ± 3.8	120 ± 7.7	**100 ± 5.2**	**130 ± 4.2**	130 ± 6.4	130 ± 5.9	**100 ± 5.1**	**115 ± 3.8**	109 ± 5.0	120 ± 5.0	**100 ± 15.2**	**145 ± 11.4**	143 ± 10.7	147 ± 22.8

*Hla-dmb*	**100 ± 4.6**	**137 ± 4.6**	133 ± 5.2	140 ± 7.3	**100 ± 3.9**	**137 ± 3.7**	139 ± 5.9	135 ± 4.9	**100 ± 7.4**	**126 ± 4.4**	115 ± 6.6	133 ± 4.7	**100 ± 3.3**	**164 ± 8.3**	157 ± 13.7	172 ± 8.4

*Hla-dra*	**100 ± 7.9**	**404 ± 19.0**	418 ± 28.1	395 ± 26.5	**100 ± 14.7**	**302 ± 23.4**	325 ± 47.0	285 ± 23.4	**100 ± 9.7**	**181 ± 22.4**	190 ± 43.4	175 ± 26.4	**100 ± 24.0**	**630 ± 51.8**	676 ± 67.9	575 ± 79.8

*Hsbp1*	**100 ± 12.8**	**168 ± 9.6**	165 ± 14.0	170 ± 13.6	**100 ± 8.7**	**209 ± 11.0**	225 ± 18.7	197 ± 13.0	**100 ± 9.5**	**109 ± 11.5**	80 ± 14.2	130 ± 13.8	**100 ± 17.7**	**287 ± 25.1**	313 ± 30.1	258 ± 40.9

*Icam1*	**100 ± 5.5**	**155 ± 7.6**	168 ± 11.9	146 ± 9.4	**100 ± 5.6**	**143 ± 4.2**	164 ± 13.3	131 ± 5.7	**100 ± 12.2**	**102 ± 6.5**	100 ± 13.5	102 ± 6.5	**100 ± 11.1**	**146 ± 11.8**	145 ± 16.7	147 ± 18.3

*Lgals3*	**100 ± 4.9**	**264 ± 16.6**	262 ± 21.4	266 ± 24.9	**100 ± 7.3**	**198 ± 12.6**	218 ± 16.3	184 ± 17.4	**100 ± 10.5**	**184 ± 12.9**	163 ± 10.0	200 ± 20.2	**100 ± 21.4**	**342 ± 34.1**	368 ± 54.6	303 ± 39.2

*Hla-drb1*	**100 ± 11.0**	**713 ± 57.0**	712 ± 96.9	713 ± 73.8	**100 ± 9.7**	**439 ± 34.8**	477 ± 73.9	412 ± 30.1	**100 ± 14.1**	**291 ± 40.2**	225 ± 44.4	342 ± 59.0	**100 ± 7.6**	**1050 ± 67.3**	1070 ± 97.4	1026 ± 100.2

*S100a6*	**100 ± 2.8**	**203 ± 11.5**	200 ± 16.2	206 ± 16.9	**100 ± 7.5**	**221 ± 13.4**	248 ± 20.9	202 ± 15.5	**100 ± 16.3**	**110 ± 8.3**	88 ± 15.5	123 ± 7.4	**100 ± 9.0**	**152 ± 13.1**	161 ± 24.3	145 ± 15.5

*Tlr2*	**100 ± 6.3**	**106 ± 5.7**	109 ± 9.8	104 ± 7.2	**100 ± 11.2**	**154 ± 9.6**	166 ± 18.9	146 ± 9.7	**100 ± 16.6**	**172 ± 18.8**	166 ± 36.7	176 ± 22.7	**100 ± 13.0**	**164 ± 11.3**	155 ± 19.2	173 ± 12.9

*Tlr4*	**100 ± 2.7**	**135 ± 8.1**	118 ± 12.8	143 ± 9.7	**100 ± 8.4**	**196 ± 9.5**	200 ± 19.7	193 ± 9.6	**100 ± 8.7**	**119 ± 4.0**	118 ± 5.9	120 ± 5.7	**100 ± 8.3**	**151 ± 10.1**	141 ± 4.9	160 ± 19.7

*Tlr7*	**100 ± 3.5**	**134 ± 6.5**	134 ± 17.2	135 ± 3.9	**100 ± 8.1**	**173 ± 5.0**	174 ± 9.6	172 ± 5.7	**100 ± 11.6**	**94 ± 7.6**	95 ± 11.6	93 ± 10.7	**100 ± 13.3**	**169 ± 11.3**	165 ± 16.6	173 ± 16.5

### Hippocampal expression of astrocytic and microglial markers is upregulated with aging

**Figure 3:**
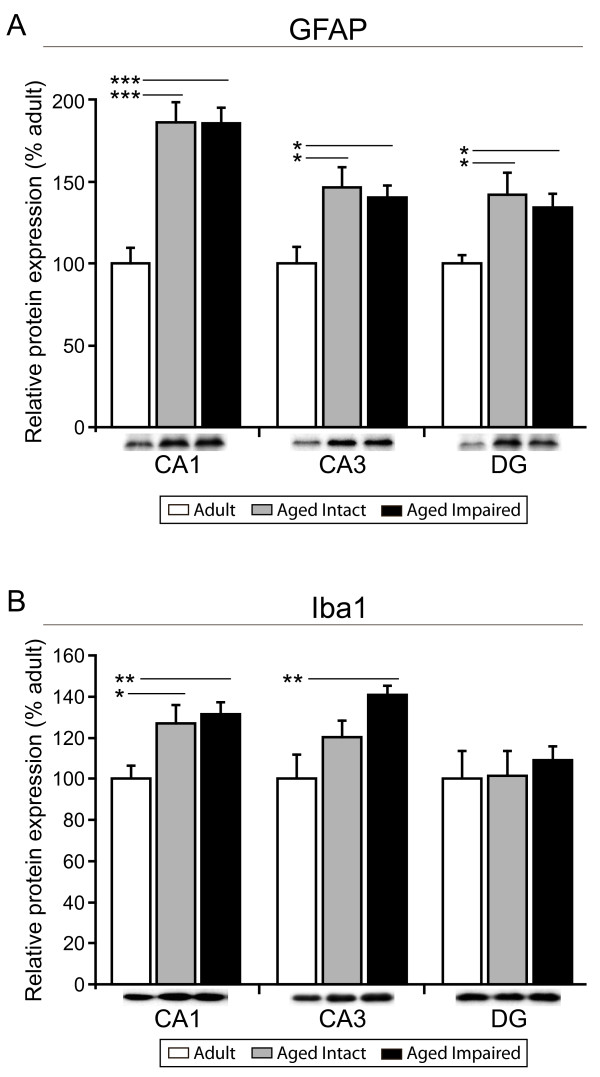
**Age-related upregulation of hippocampal astrocyte and microglial activation protein markers**. (A) Immunoblotting for GFAP, an intermediate filament protein expressed by astrocytes and upregulated with inflammation, is significantly increased in CA1, CA3 and DG of both aged cognitively intact and aged cognitively impaired rats compared to adults. (B) Iba1, a microglia-specific calcium binding and actin-bundling protein, is significantly induced in CA1 and CA3, but not DG of aged animals, regardless of cognitive status, compared to adults. Insets depict representative immunoblot images. *p < 0.05, **p < 0.01, ***p < 0.001, one-way ANOVA with Student Newman Keuls post hoc testing, n = 7-10/group.

Upregulation of MHC II-associated genes with aging suggests a heightened immune response [45] in the aged hippocampus. To determine whether increased expression of these genes is associated with age- or cognitive status-related increases in glial activation, and whether potential glial activation occurs throughout the hippocampus or in a circumscribed subregion, expression of astrocyte-specific (GFAP) and microglia-specific (Iba1) protein markers was assessed by immunoblotting in adult, aged cognitively intact, and aged cognitively impaired rats. In both aged intact and aged impaired rats, significant increases in GFAP expression ranging from 50% to 80%, relative to adults, were detected in CA1 (p < 0.001), CA3 (p < 0.05) and DG (p < 0.05) (ANOVA, SNK; Figure 3A). Likewise, Iba1 expression was significantly elevated by ~30% in CA1 of aged intact (p < 0.05) and aged impaired (p < 0.001) rats compared to adults, and in CA3 of aged impaired rats compared to adults (~45%, p < 0.001) (ANOVA, SNK; Figure 3B). A similar trend was observed in the CA3 of aged intact rats, although it did not reach statistical significance. No changes in expression of Iba1 were detected in DG. Expression of GFAP and Iba1 was not different between aged intact and aged impaired rats in any subregion and did not correlate to individual animal MWM performance.

### Immunohistochemical visualization of astrocyte activation

**Figure 4:**
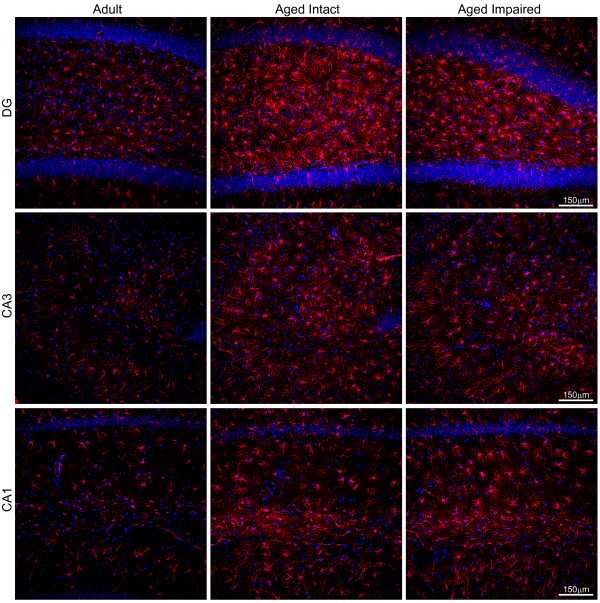
**Age-induced activation of hippocampal astrocytes**. Immunohistochemical visualization of GFAP revealed low-to-moderate expression in astrocytes distributed throughout DG, CA1 and CA3 of adults. GFAP immunoreactivity was markedly increased, and astrocyte morphology indicated activation, throughout the hippocampal formation of aged rats. No qualitative differences between aged intact and aged impaired rats were evident, and no subregion-specific alterations in astrocyte activation were observed. Increased GFAP immunoreactivity was associated with astrocyte hypertrophy, but not astrocyte proliferation, as total populations of GFAP^+ ^glia were not changed between groups in CA1, CA3 or DG. Blue: Hoechst; Red: GFAP.

To extend immunoblot data indicative of glial activation, immunohistochemical characterization of astrocytes was performed using adult, aged intact, and aged impaired rats. This approach enabled visualization of age-related changes in GFAP immunoreactivity as well as assessment of potential changes in localization of astrocytes associated with aging and cognitive impairment (Figure 4). GFAP-immunoreactive astrocytes were distributed throughout DG, CA1 and CA3, and were associated with both cell body and synaptic layers. Notable increases in the intensity of GFAP immunoreactivity within cells was evident in CA1 and CA3 of aged cognitively intact and aged cognitively impaired rats compared to adults, with the most dramatic age-related changes evident in DG. No subregion-specific qualitative differences in astrocyte immunoreactivity or distribution were evident between aged intact and aged impaired rats, suggesting that increased astrocyte activation is associated with advanced age but not further exacerbated with cognitive decline. Quantitation of GFAP^+ ^cells revealed no age- or cognitive decline-associated changes in astrocyte density (mean ± S.E.M) in DG (astrocytes/100 μm^2^: adult: 60 ± 2.7, aged intact: 58 ± 1.1, aged impaired: 61 ± 1.8), CA3 (astrocytes/100 μm^2^: adult: 74 ± 2.4, aged intact: 76 ± 3.2, aged impaired: 72 ± 1.2), or CA1 (astrocytes/100 μm^2^: adult: 56 ± 1.3, aged intact: 54 ± 1.5, aged impaired: 56 ± 2.1). These results suggest that the age-related increase in GFAP expression demonstrated by immunoblotting and immunohistochemistry reflects increased astrocyte activation rather than proliferation.

**Figure 5:**
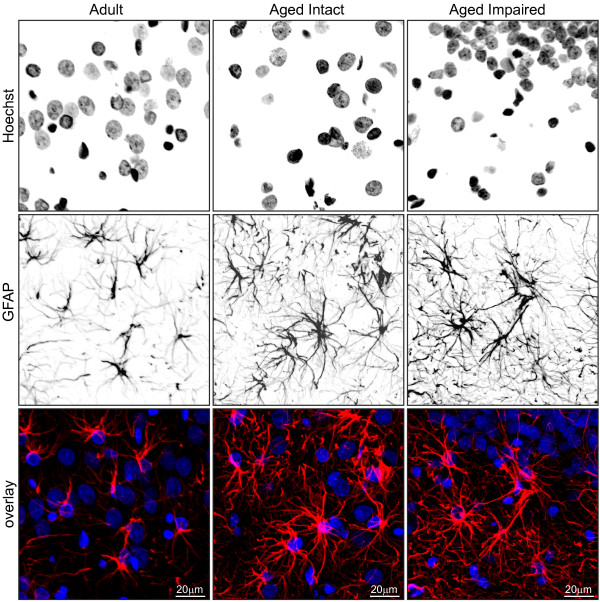
**Morphological characteristics of activated astrocytes in aged hippocampus**. Higher magnification imaging revealed a stellate morphology and low level of GFAP immunoreactivity in hippocampal astrocytes of adult rats. In hippocampus of both cognitively intact and cognitively impaired aged rats, astrocytes exhibited intense GFAP immunoreactivity and adopted a highly ramified morphology with hypertrophic processes, while maintaining spatial compartmentalization.

Morphological assessment also revealed an activated astrocytic phenotype associated with the aged hippocampus (Figure 5). In adult rats, astrocytes expressed low levels of GFAP, exhibited a stellate morphology with numerous thin, branched processes, and were spatially distinct. In both aged intact and aged impaired rats, cellular GFAP immunoreactivity was dramatically increased and astrocytes appeared hypertrophic. Consistent with mild reactive gliosis, GFAP-containing processes were visibly thicker and more highly ramified, and a degree of spatial overlap was evident.

### Quantitation of activated microglia

**Figure 6:**
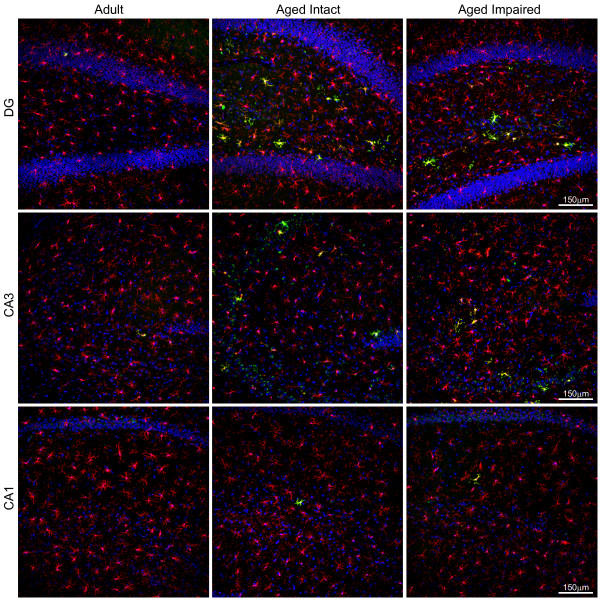
**Activation of hippocampal microglia with aging**. Immunohistochemical co-localization of the microglia-specific marker Iba1 and the activation-specific marker CD74 (MHC II invariant chain) identified an increase in activated microglia in the aged hippocampus regardless of cognitive status. In adults, Iba1^+ ^microglia were distributed throughout hippocampal subregions, with a greater population evident in CA3 than in CA1 or DG. A small fraction of microglia exhibited mild activation indicated by low levels of CD74 immunoreactivity. In both aged cognitively intact and aged cognitively impaired rats, a marked increase in the number of Iba1^+^/CD74^+ ^microglia was observed in all three subregions, in the form of both mildly-activated and moderately-activated microglia. Activated microglia were associated with both synaptic and cell body-containing hippocampal strata. Blue: Hoechst; Red: Iba1; Green: CD74; Orange: Iba1/weak CD74 co-expression (mild activation); Yellow: Iba1/high CD74 co-expression (moderate activation).

Visualization of activated microglia was achieved by immunohistochemical co-detection of Iba1 (microglia-specific marker) and CD74 (OX-6 antibody, MHC II invariant chain; activation-specific microglial marker) (Figure 6). Co-staining offers the benefit of determining the percentage of total microglia activated as opposed to measuring CD74 alone. Iba1-immunoreactive microglia were evenly distributed throughout DG, CA1 and CA3 in adult rats. Few CD74^+ ^activated microglia were observed in adult hippocampi. In both aged cognitively intact and aged cognitively impaired rats, increased Iba1 immunoreactivity was apparent, as was a marked increase in the appearance of activated (Iba1^+^/CD74^+^) microglia. CD74-imunoreactive cells were associated with both synaptic and cell body (granule/pyramidal) layers throughout all three hippocampal subregions.

**Figure 7:**
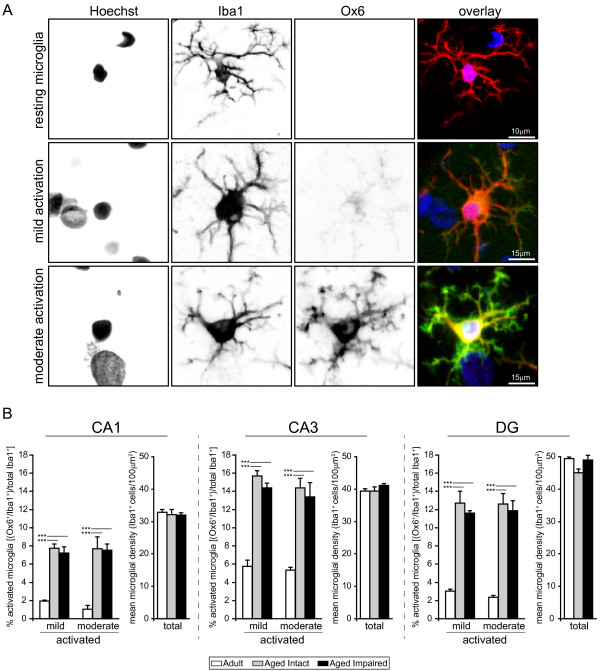
**Age-related increase in activated microglial populations**. (A) High-magnification visualization of resting/surveilling, or non-activated, and activated microglia reveals differences in both immunoreactivity and morphology with aging. Non-activated microglia expressed Iba1 but not CD74, and had long, thin, branched processes. Microglia undergoing mild activation displayed increased Iba1 immunoreactivity, weak CD74 immunoreactivity, and enlarged somata, while moderately activated microglia demonstrated increased Iba1 immunoreactivity, intense CD74 immunoreactivity, enlarged somata, thickened proximal processes, and retracted distal processes. Notably, activated microglia maintained a ramified rather than ameboid morphology, indicating that microglial activation had not progressed to a reactive, phagocytic phenotype. (B) Quantitation of activated microglia (calculated as the ratio of Iba1+/CD74^+ ^to total Iba1^+ ^cells) revealed significant increases in all three hippocampal subregions studied. In adult rats, a small fraction of total microglia were mildly or moderately activated in CA3, with far fewer activated microglia evident in CA1 and DG. The percentage of microglia undergoing mild or moderate activation was significantly increased, by several fold, throughout the hippocampus of both aged cognitively intact and aged cognitively impaired rats compared to adults. No differences between the degree of microglial activation (*i.e*., mild versus moderate) were observed between aged intact and aged impaired rats in CA1, CA3 or DG. Increased numbers of activated microglia in aged rats were not due to proliferation/infiltration, as total populations of microglia (Iba1^+ ^cells) were not different between groups. ***p < 0.001, one-way ANOVA with SNK post hoc testing.

Higher magnification of microglia revealed changes in both protein expression and morphological characteristics of activation (Figure 7A). Resting microglia were immunoreactive to Iba1 but not CD74, and had numerous thin, branched projections. Activated microglia expressing both Iba1 and CD74 exhibited characteristics of both mild activation (increased Iba1 immunoreactivity, weak CD74 immunoreactivity, enlarged somata) and moderate activation (increased Iba1 immunoreactivity, intense CD74 immunoreactivity, enlarged somata, thickened proximal processes, retracted distal processes). The percentage of mildly- and moderately-activated microglia relative to total microglia was calculated in each subregion of adult, aged intact, and aged impaired rats (Figure 7B). The percentage of activated microglia in adult rats varied by subregion, with microglia in states of mild or moderate activation reflecting 1-2% of total microglia in CA1, 6% in CA3, and 2-3% in DG. Significant age-related increases in both mildly-activated and moderately activated microglia were evident in all three subregions of both cognitively intact and cognitively impaired aged rats compared to adults (p < 0.001, ANOVA, SNK), with no differences in either mild or moderate activation between aged intact and aged impaired groups. In CA1 of both aged intact and aged impaired rats, the percentage of both mildly- and moderately-activated microglia increased by approximately 8-fold, to 7-8% of total microglia. In CA3 of aged rats, 13-16% of microglia reflected either mild or moderate activation regardless of cognitive status, representing a 2.5-fold increase in activation compared to adults. In DG of both aged intact and aged impaired rats, mild and moderate microglial activation increased by 6-fold (12-13% of total microglia) compared to adults. No correlation between the percentage of activated microglia (mild or moderate) and MWM performance was observed. No differences in the proportion of mildly-activated to moderately-activated microglia were observed between groups in CA1, CA3 or DG. The density of microglia was the same (Iba1^+ ^cells/100 μm^2^) across groups (Figure 7B) indicating that the increase in activated microglial does not reflect proliferation or infiltration, but rather that an increased percentage of the existing microglial population is activated in the aged hippocampus.

**Figure 8:**
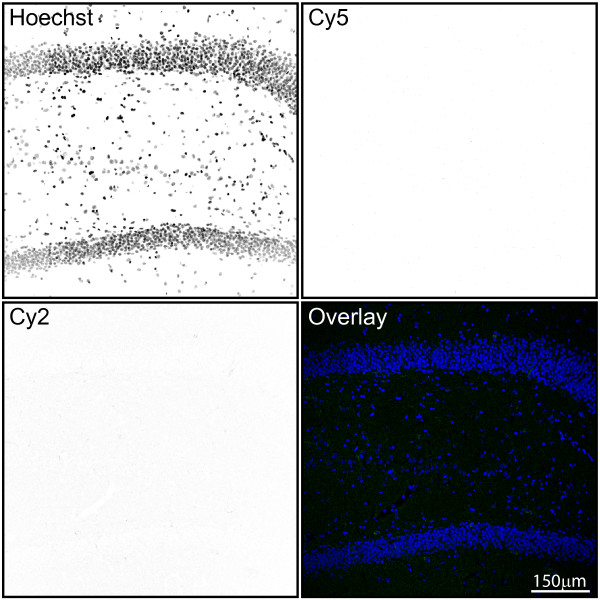
**Assessment of negative control signals in immunohistochemical experiments**. To ensure that visualization of astrocytes and microglia was not confounded by nonspecific secondary antibody binding, a negative control for the Cy2 (donkey-anti-mouse DyLight 488) and Cy5 (donkey-anti-rabbit DyLight 649) channels was included with primary antibodies omitted. Individual channels are presented as inverted gray scale. Weak, diffuse background signal was observed in the Cy2 channel, and no signal was apparent in the Cy5 channel. No nonspecific cell staining in the absence of primary antibodies occurred. Blue: Hoechst; Green: Cy2; Red: Cy5.

Examination of the negative control slides incubated with secondary antibodies with primary antibodies omitted demonstrated that immunoreactivity to GFAP, Iba1, and CD74 was antigen-specific, as no non-specific background signal was detected on these controls (Figure 8).

## Discussion

Neuroinflammation (*i.e.*, expression of inflammatory response factors and glial activation), is a hallmark of brain aging [46,47] and is implicated in the cognitive deficits that accompany neurodegenerative conditions and profound brain insults (*e.g*., TBI and sepsis) [48-50]. Markers of neuroinflammation (*e.g*., CD74, GFAP) have been reported to increase in the hippocampus with age but the manner in which these changes may contribute to cognitive decline with normative aging remain to be determined. Here, we demonstrate that a coordinated induction of MHC II immune response-associated genes and concomitant astrocyte/microglial activation in the hippocampus occurs with advanced aging in both cognitively intact and impaired animals but does not correlate with age-related deficits of cognitive function.

In this work, bioinformatic analysis of the hippocampal synaptosomal transcriptomes of adult and aged rats behaviorally assessed for spatial learning and memory identified 21 genes as components of an age-induced MHC II-associated antigen presentation and response pathway. Upregulation of these genes with advanced aging was confirmed by qPCR in whole-hippocampus synaptosome fractions and in hippocampal subregion (CA1, CA3 and DG) dissections. In agreement with our findings, examination of primary microarray datasets from previous transcriptomic studies of age-related changes in hippocampal gene expression from humans [51], non-human primates [52], and rodents [53,54] reveals upregulation of a number of immune response factors, including induction of MHC II-associated genes, that have not previously been systematically pursued in follow-up studies. For example, transcriptomic analyses of Fischer 344 rat hippocampal gene expression reveals age-related upregulation of MHC II alpha chain, Cd74, Fcgr3, and C3 [55], as well as induction of MHC II beta and invariant chains and multiple complement components [56]. Importantly, no statistical relationships between age-related increases in these specific inflammation-response genes and cognitive performance were identified in these studies. Increased Cd74, C4a and C3 mRNA expression, which we observed in aged rats compared to 12-month old adults, has also been observed in the hippocampal transcriptome between young (4-6 months) and aged (24 months) Fischer 344 rats [57].

Our findings also share commonalities with previous targeted gene expression studies of hippocampal aging. Increased hippocampal expression of Hla-dra has been reported in aged (24 months) versus young (3 months) Fischer 344 × Brown Norway rats [58]. We have expanded on this work by demonstrating that increased expression of multiple MHC II components [*i.e*., MHC II alpha (Hla-dra), beta (Hla-drb1) and invariant (Cd74) chains, and the MHC II antigen-loading cofactor (Hla-dmb)], and multiple MHC II pathway-associated genes occurs between mature adult (12 months) and aged (26-28 months) rats. Similarly, the present study builds upon a previous finding of increased toll-like receptor (TLR) expression with aging in mouse whole-brain preparations [59], by demonstrating that increased expression of TLRs 3, 4, and 7 occurs primarily in hippocampal synapses and in the hippocampal CA3 subregion, and that TLR expression does not correlate with cognitive impairment.

Our findings extend these previous reports by characterizing a large set of MHC II components and associated immune/inflammation response factors and by demonstrating that increased expression of these genes occurs in both cognitively intact and impaired animals but that the extent of induction does not correlate with cognitive deficits. Interestingly, the magnitude of induction of these MHC II-mediated immune response genes was greater in CA1 and CA3 than in DG in nearly all cases, suggesting that pyramidal cell-containing subregions may undergo age-related neuroinflammation to a greater extent than granule cell-containing regions. Further, age-related increases in immune/inflammation response gene expression were, in many cases, larger in hippocampal synaptosome fractions than in dissected hippocampal subregions. MHC II pathway gene expression in hippocampal synaptosomes likely stems largely from astrocytic and microglial processes closely associated with synaptic compartments, while downstream inflammatory signaling factors may derive from the synaptic terminals themselves. This suggests that heightened antigen processing/immune response within the synaptic milieu may contribute to age-related dysfunction of hippocampal synapses and potentially plays a role in synapse loss [60-62].

Immunohistochemical studies of both human and nonhuman primate aging have demonstrated upregulation of MHC II alpha/beta chains in hippocampal microglia [18,21,63,64]. We have observed, in both aged cognitively intact and aged cognitively impaired rats, widespread mild and moderate microglial activation indicated by increased numbers of microglia expressing CD74 (*i.e*., the MHC II invariant chain), and altered morphological and immunoreactive phenotypes. While the percentage of activated microglia (reflecting the ratio of CD74^+^/Iba1^+ ^to total Iba1^+ ^microglia) was consistently increased by several fold in all aged rats compared to adults, these glia maintained a ramified morphology, rather than the ameboid, morphology typical of a phagocytic phenotype. This suggests that the neuroinflammatory state in the aged hippocampus, while elevated compared to adults, is not severe enough to induce a transition from a "surveilling" microglial phenotype [65,66] to the reactive phenotype associated with dramatic CNS insults and neurodegeneration [66-68]. In agreement with previous work, the total number of microglia (Iba1^+ ^cells) did not increase with aging [69], indicating that increases in Iba1 protein expression and numbers of CD74+ microglia reflect age-related activation rather than microglial proliferation or infiltration. Iba1/CD74 co-labeling extends previous findings of increased hippocampal microglial activation (CD74+ only) in comparisons of young (3-6 months) and aged (24+ months) [70,71] by demonstrating that an increased percentage of total hippocampal microglia are activated with advanced aging compared to mature adults. Additionally, previous studies [70-72] have generally compared young (3-6 months) and old (24+ months) rats, while our results demonstrate that microglial activation occurs between mature adulthood (12 months) and advanced age (24-26 months). Furthermore, we extend a previous finding of qualitative differences in populations of activated microglia in the aged Wistar rat hippocampus [70] by demonstrating that increased activation is evident across CA1, CA3, and DG, and that, within each subregion, the percentages of qualitatively distinct activated microglia (mild and moderate) are equivalent. Similar to the work by Gavilan and colleagues [70], fewer activated microglia were observed in close proximity to pyramidal and granule cell layers than associated with synapse-containing hippocampal layers. While adding to the evidence of hippocampal microglial activation with advanced aging, our findings also clearly demonstrate that the examined marker of microglial activation (CD74) is not directly associated with deficits of spatial learning and maze performance, in agreement with previous studies in the Long Evans rat [72].

We also observed increased astrocyte activation, as indicated by increased GFAP expression and morphological alterations, which occurred in the absence of astrocyte proliferation. Studies of humans, nonhuman primates, and rodent models of human aging have demonstrated increased glial fibrillary acidic protein (GFAP) gene and protein expression indicative of astrocyte activation and age-related astrocyte hypertrophy, in agreement with our findings [73-79]. Our observations agree with one of the first investigations of age-related hippocampal astrocyte activation, which demonstrated that these dystrophic astrocyte processes are often oriented in the same direction [77]. Our work also revealed that while astrocyte activation increases throughout the hippocampus with advanced aging, no cognitive decline-associated differences in GFAP protein content or numbers of GFAP^+ ^astrocytes are apparent between aged intact and aged impaired rats.

Our finding that MHC II pathway induction and glial activation occur in aged rats regardless of cognitive status raises the important question of whether neuroinflammation plays a role in the pathogenesis of cognitive decline [47]. Pathological gliosis and inflammation are associated with severe cognitive dysfunction in neurodegenerative/advanced disease states, traumatic brain injury, and direct inflammatory stimulation [17-24]. Hippocampal neuroinflammation has also been suggested to play a functional role in the etiology of age-related cognitive impairment [80,81]. A lesser degree of neuroinflammation occurs with normative aging than with disease or injury, and ranges from increased oxidative stress to induction of inflammatory signal transducing factors including cytokines, chemokines, complement, and stress response proteins which we and others have previously described in studies of hippocampal aging [12,25,27,55,82-84]. Age-related alterations in these factors suggest an allostatic shift toward a heightened basal neuroinflammatory state in the aging hippocampus. Establishment and maintenance of such a state has been termed "para-inflammation" and is thought to represent an adaptive response to persistent sub-threshold stimuli such as cellular/tissue stress [45].

The present work demonstrates that, while neuroinflammation at the level of MHC II pathway expression and glial activation may represent an aspect of age-related hippocampal dysregulation necessary for development of cognitive deficits, these measures do not correlate to cognitive performance deficits. It is likely that multiple age-related processes, including decreased neurotransmission, glial dysfunction, and increased neuroinflammation, combine with cognitive decline-related dysregulation of plasticity- and myelin-associated proteins and genes [27,55], to form an additive array of insults to impede healthy neuronal function and learning and memory [3]. Thus far, the triggering event(s) that cause the transition from an aged, cognitively intact state to an aged, cognitively impaired state have remained elusive. Additionally, the potential remains that other measures of neuroinflammation, including cytokines in both the CNS and systemically could directly correlate to impaired spatial learning and memory. The importance of the interplay between systemic inflammation, neuroinflammation, and brain function with aging was recently underscored by a report demonstrating inhibition of hippocampal neurogenesis by the systemic cytokine CCL11 [85]. Ultimately, our understanding of the functional roles of neuroinflammation in both protective and harmful CNS processes must advance in order to identify whether there are appropriate points for interventions seeking modulate age-related neuroinflammation with the goal of preventing or reversing age-related hippocampal dysfunction.

## Conclusions

These data demonstrate the age-related induction of mild to moderate neuroinflammation with aging in multiple regions of the hippocampus. Analysis of neuroinflammatory measures (MHC II pathway-associated gene expression, and astrocyte and microglial activation) in rats assessed for spatial learning and memory by Morris water maze testing revealed a lack of correlation between inflammation and behavioral performance, suggesting that these aspects of hippocampal neuroinflammation alone are not sufficient to induce age-related impairment of spatial learning and memory. Future work building on this demonstration of age-related induction of MHC II pathway and inflammatory response factor expression and glial activation in both cognitively intact and cognitively impaired aged animals will need to examine if and how age-related hippocampal changes such as those described here combine with cognition-specific alterations to ultimately impair spatial learning and memory.

## Abbreviations

CA1: cornu ammonis 1; CA3: cornu ammonis 3; DG: dentate gyrus; MHC II: major histocompatibility complex class II; CLIP: class II-associated invariant chain peptide; GFAP: glial fibrillary acidic protein; Iba1: ionized calcium binding adaptor molecule 1: SNK: Student Newman Keuls; BHMTC: Benjamini-Hochberg multiple testing correction

## Competing interests

The authors declare that they have no competing interests.

## Authors' contributions

HDV, WMF, and WES conceived and designed the studies. JAF conducted behavioral testing, and HY and JPW performed hippocampus dissections. HDV, GVB, RMB executed and analyzed the experiments. HDV and WMF wrote, and all authors edited, read, and approved the final manuscript.
